# Precipitation Behavior and Quenching Sensitivity of a Spray Deposited Al-Zn-Mg-Cu-Zr Alloy

**DOI:** 10.3390/ma10091100

**Published:** 2017-09-19

**Authors:** Xiaofei Sheng, Qian Lei, Zhu Xiao, Mingpu Wang

**Affiliations:** 1 School of Materials Science and Engineering, Central South University, Changsha 410083, China; auden1@126.com (X.S.); xiaozhumse@163.com (Z.X.); wangmp@csu.edu.cn (M.W.); 2 School of Materials Science and Engineering, Hubei University of Automotive Technology, Shiyan 442002, China; 3 Department of Materials Science and Engineering, College of Engineering, University of Michigan, Ann Arbor, MI 48109, USA

**Keywords:** spray deposition, microstructure, aluminum alloys, strength, precipitation

## Abstract

Precipitation behavior and the quenching sensitivity of a spray deposited Al-Zn-Mg-Cu-Zr alloy during isothermal heat treatment have been studied systematically. Results demonstrate that both the hardness and the ultimate tensile strength of the studied alloy decreased with the isothermal treatment time at certain temperatures. More notably, the hardness decreases rapidly after the isothermal heat treatment. During isothermal heat treatment processing, precipitates readily nucleated in the medium-temperature zone (250–400 °C), while the precipitation nucleation was scarce in the low-temperature zone (<250 °C) and in the high-temperature zone (>400 °C). Precipitates with sizes of less than ten nanometers would contribute a significant increase in yield strength, while the ones with a larger size than 300 nm would contribute little strengthening effect. Quenching sensitivity is high in the medium-temperature zone (250–400 °C), and corresponding time-temperature-property (TTP) curves of the studied alloy have been established.

## 1. Introduction

In the past decades, 7xxx series (Al-Zn-Mg-Cu) aluminum alloys exhibited an outstanding performance and attracted much interest in many industry fields, especially in the automotive and the aviation field [1,2,3], because of their high strength, high ductility, and low density [4,5]. The high strength makes the products strong, and the high ductility allows the products to be easily forming during the machining process, and the low density saves the energy cost and reduces the carbon emission. Rolling, extrusion, drawing, and other conventional thermo-mechanical processing have limited capabilities to improve the strength of Al-Zn-Mg-Cu alloys [6,7,8,9,10]. To achieve a higher strength, some new technologies have been developed recently [11,12,13,14]. Severe plastic deformation (SPD) techniques, such as accumulative roll bonding [11], high-pressure torsion [12], equal channel angular pressing [13], and friction stir processing [14], were proven to be effective methods to refine the structure and enhance the strength. However, SPD techniques yield laboratory-scale samples, and they are still rare in industry [15]. Materials with fine grains usually exhibit a high strength (due to the Hall-Petch relation) [16,17,18,19,20,21,22]. The spray deposition technology has been developed rapidly [23,24]. Large-scale Al-Zn-Mg-Cu-Zr ingots with fine structure have been successfully fabricated by the spray deposition technology in our previous work [25,26]. The obtained ingots contain fine grains due to the rapid solidification and alloying [27,28,29]. Most investigations focus on the microstructure and properties of the spray-deposited Al-Zn-Mg-Cu alloy in the lab scale [26,30]. Thermal heat treatment is an efficient method to enhance the properties. However, some critical process parameters should be reconsidered when the large ingots were turned to industrial products. For example, the solid solution-quenching treatment for precipitation hardening alloys is a very significant process, which would affect the followed aging process and the final properties [31]. In industry, ingots are about one ton in weight and a half cubic meter in volume, which are difficult to quench as fast as a sample with a lab-scale size. Decomposition will occur during the cooling down process. In addition, microstructure evolutions, precipitation behavior, properties variation, and quenching sensitivity of a spray deposited Al-Zn-Mg-Cu alloy during isothermal heat treatment (IHT) have not been reported. In this work, large-scale Al-Zn-Mg-Cu-Zr ingots were synthesized by a spray deposition technology. Microstructure evolution and properties variation of the solid solution treated alloy during isothermal heat treatments were investigated systematically. Precipitation behavior, quenching sensitivity, and strengthening mechanisms have been discussed.

## 2. Materials and Methods

The studied alloy was synthesized with an SFZD-5000 type environmental chamber in Haoran company (Xinghua, China) [25,26]. The chemical compositions of the spray-deposited Al-Zn-Mg-Cu-Zr alloy are listed in Table 1. Before the spray deposition process, blocks of Al, Zn, Mg, Cu, and Al-Zr master alloy were melted, and then the molten melt was atomized by N_2_ gas. The distance of the atomizing deposition was 650 mm, rod ingots were spray deposition formed with a size of 500 mm in diameter and 1800 mm in length. The as-deposited ingot was extruded at 420 °C with an extrusion speed of 3 mm/s and an extrusion ratio of 6.25:1 (500 mm to 200 mm in diameter). Experimental specimens were cut from the hot extruded rod, and they were solid solution treated at 470 °C/1 h in the first furnace with a melted mixture of 50% NaNO_3_ and 50% KNO_3_, and then rapidly transferred into another muffle furnace with a certain temperature (205–445 °C) for isothermal heat treatment. Some of the isothermal heat treated specimens were aged at 120 °C for 24 h. Table 2 gives the detailed parameters of the samples after different heat treatments. The sample A solid solution treated at 470 °C/1 h was used as a reference set.

Hardness measurements of heat treated specimens were conducted on a HV-5 Vickers hardness tester (Laizhou Huayin Testing Instrument Co., Ltd., Laizhou, China) to keep track of the hardness variation with the aging time. The load is 2 kg, the dwell time is 15 s, and each average hardness was taken from at least seven measurements. Flat-shoulder tensile test samples under the same condition were prepared with dimensions of a gage length of 25 mm, and a width of 10 mm. Tensile testing experiments were performed on an MTS 810 material testing machine (MTS Systems Corporation, Eden Prairie, MN, USA) at room temperature with a strain rate of 2 mm/min. Microstructure observations on the isothermal heat treatment specimens were operated on an FEI Tecnai G^2^ F20 transmission electron microscope (TEM) (FEI Company, Hillsboro, OR, USA) with an operation voltage of 200 kV. All TEM specimens were prepared by mechanical grinding, and then polished by a double-jet electropolishing device (MTP-1A) (Shanghai Jiao Da Electrical and Mechanical Technology Development Co., Ltd., Shanghai, China) at −30 °C with a solution of 30% nitric acid +70% methanol.

## 3. Results

### 3.1. Microstructure Evolutions during Isothermal Heat Treatment and Aging

**Figure 1:**
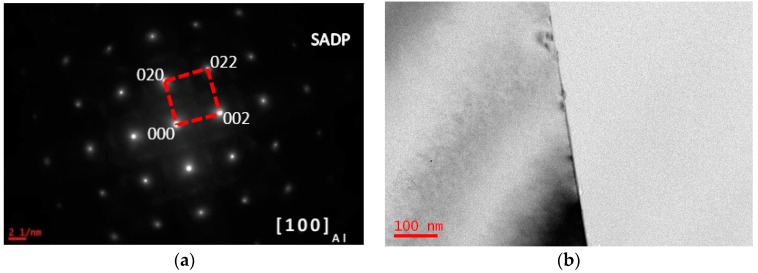
Microstructure and selected area diffraction patter (SADP) of the sample having been solution treated at 470 °C for 1 h. (**a**) SADP of the sample, beam along to zone axis of [100]_Al_; (**b**) bright-field image.

For the as-spray deposited sample, there is no dendrite and segregation due to rapid solidification, and smaller grain size were detected in the spray deposited ingot, in comparison to that of the conventional casting ingots [32]. Bright-field (BF) images and selected area diffraction pattern (SADPs) of the studied alloy (sample A) having been solid solution treated at 470 °C/1 h and quenched with water with a quenching rate of 200 °C/s are shown in Figure 1. After solid solution treatment, metallic compounds precipitated during spray deposited process were re-dissolved into the matrix and a supersaturated solid solution (SSS) formed. The SADP in Figure 1a shows that a set of diffraction spots from Al were detected. The micrographs of the grain interior and grain boundary show that a SSS was formed after solid solution treatment (Figure 1b).

**Figure 2:**
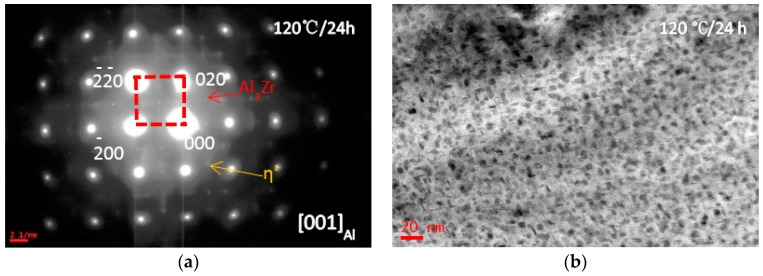
Bright-field (BF) images of the studied alloy having been solid solution treated at 470 °C for 1 h and then directly aged at 120 °C for 24 h. (**a**) SADP, the beam along zone axis of [100]_Al_; (**b**) bright-field image.

The BF image and corresponding SADPs of the solid solution treated alloy (sample B) are shown in Figure 2. After aging treatment at 120 °C for 24 h, some precipitates were formed with a size of 10 nm (Figure 2b). Some diffraction spots were from the precipitates, as seen in Figure 2a. According to the crystal geometry and crystal diffraction, indexing of the SADPs showed that the rod-like precipitates were η′-MgZn_2_ phase, which has hexagonal lattice parameters of a = 0.496 nm, c = 1.402 nm [33,34]. A series of crystal orientation relationship between the matrix and η′ phase could be calculated and proved: (001) η′||(110)Al and [100]η′||[001]Al [35]. The high strength of Al-Zn-Mg-Cu alloys are due to a significant number of η′ phase precipitates [36]. Fine spherical L1_2_-Al_3_Zr precipitates mixed with rod-like η′-MgZn_2_ precipitates in Figure 1b, and SADP in Figure 1a, proved that the SSS was decomposed after aging at 120 °C for 24 h.

**Figure 3:**
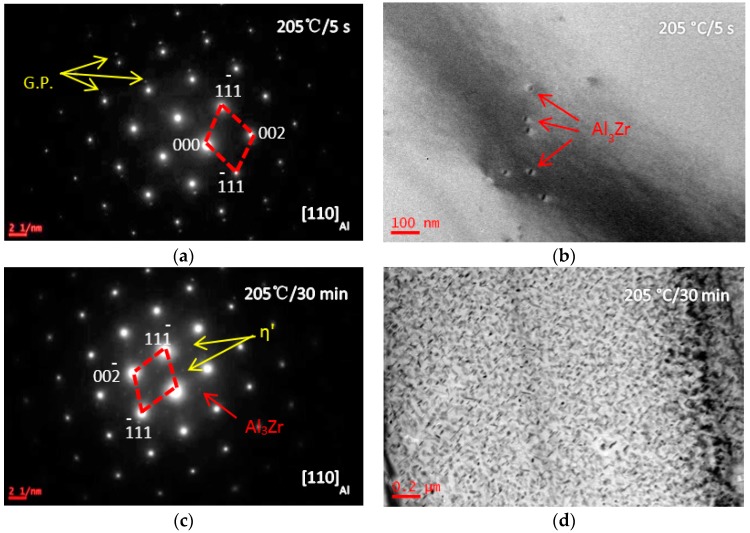
SADPs and BF images of the specimens having been isothermal heat treated at 205 °C for different durations (5 s and 30 min). (**a**) SADP of (b), beam along [110]_Al_; (**b**) the IHT time is 5 s; (**c**) SADP of (d), beam along [110]_Al_; and(**d**) the isothermal heat treatment (IHT) time is 30 min.

**Figure 4:**
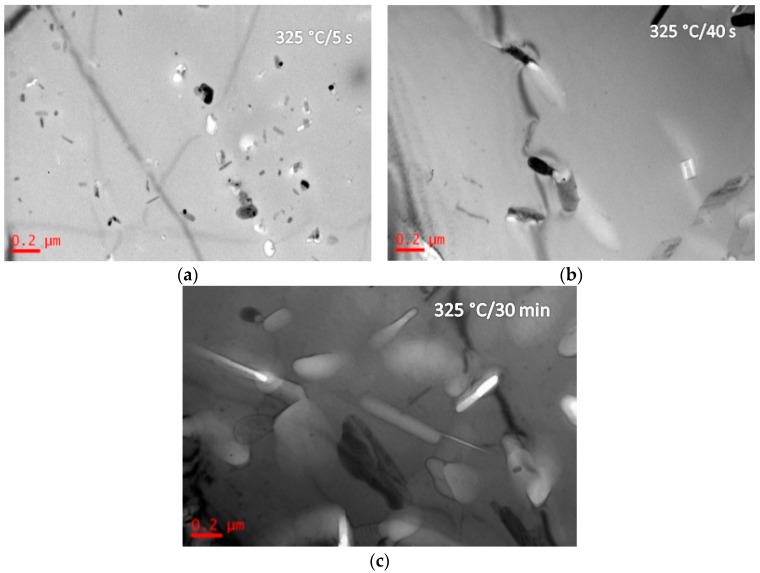
BF images of the studied alloy having been isothermal heat treated at 325 °C for different durations. The IHT durations are: (**a**) 5 s; (**b**) 40 s; and, (**c**) 30 min.

**Figure 5:**
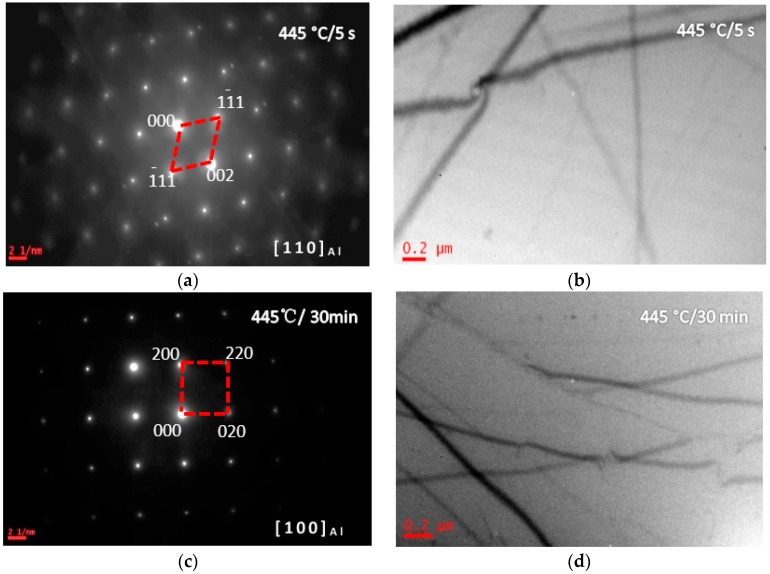
SADPs and BF images of the studied alloy having been isothermal heat treated at 445 °C for 5 s and 30 min. (**a**) SADP from the area in (b); (**b**) the isothermal heat treatment time is 5 s; (**c**) SADP from the area in (d); and (**d**) BF image, the isothermal heat treatment time is 30 min.

Figure 3 shows the BF images and SADPs of the solid solution treated alloy having been isothermal heat treated at 205 °C (quenching rate of 200 °C/s) for different durations (5 s and 30 min) (sample C). After being isothermal heat treated at 205 °C for 5 s, more diffraction spots from precipitates were detected in SADP (Figure 3a). The bright-field image of the grain inside showed G. P. zone and Al_3_Zr were formed (Figure 3b). Increasing the IHT time to 30 min, the SADP in Figure 3c presents some diffraction spots from η′ precipitates, and the SSS was decomposed, and some precipitates with a size of 80 nm in length and 10 nm in diameter were detected (Figure 3d). Figure 4 shows BF images of the studied alloys having been isothermal heat treated at 325 °C for different durations (5 s, 40 s, and 30 min) (sample D). After isothermal heat treated at 325 °C for 5 s, η′ precipitates were detected with a size of 150 nm in length and 20 nm in diameter (Figure 4a). Increasing the IHT time to 40 s, η′ precipitates were coarsened to be a size of 250 nm in length and 100 nm in diameter (Figure 4b). Further increasing the IHT time to 30 min, η′ precipitates inside grain coarsened with a size of 400 nm in length and 80 nm in diameter (Figure 4c). BF images and SADPs of the studied alloy having been isothermal heat treated at 445 °C for different durations (5 s and 30 min) (sample E) are shown in Figure 5. After isothermal heat treatment at 445 °C for 5 s even 30 min, there is few diffraction spots from precipitates were detected in the SADP (Figure 5a), and there are few precipitates (Figure 5b,c).

**Figure 6:**
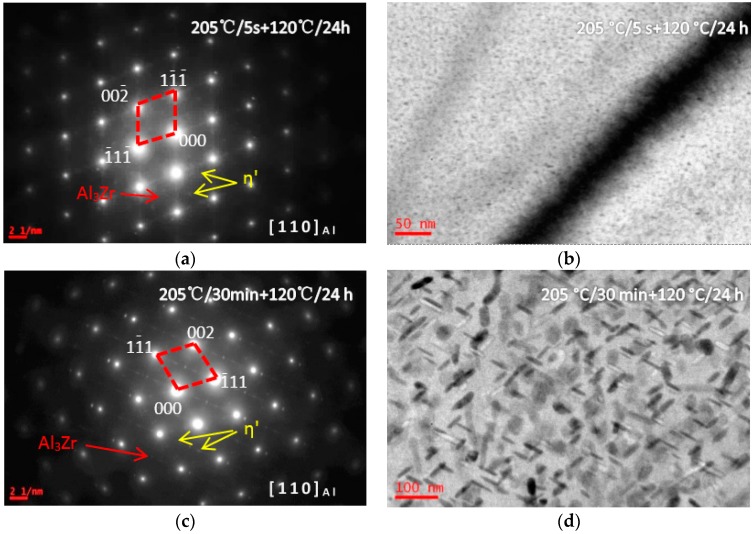
SADPs and BF images of the studied alloy having been isothermal heat treated at 205 °C for different durations (5 s and 30 min), and then aged at 120 °C for 24 h. (**a**) SADP from the area in (**b**); (**b**) the isothermal heat treatment is 5 s; (**c**) SADP from the area in (d); and (**d**) isothermal heat treatment is 30 min.

Figure 6 shows BF images and SADPs of the solution treated alloy having been isothermally heat treated at 205 °C for different durations (5 s and 30 min), and then aged at 120 °C for 24 h (sample F). After the sample was treated at 205 °C for 5 s and aged at 120 °C for 24 h, some diffraction spots from η′ and Al_3_Zr precipitates were detected in the SADP (Figure 6a), and the bright-field images of grain inside and grain boundary also shows many η′ precipitates, whose size was 20–40 nm (Figure 6b). Increasing the IHT time to 30 min, the SADP in Figure 6c presented many diffraction spots from precipitates, and many coarse η′ precipitates with a size of 100 nm in length and 10 nm in diameter were detected (Figure 6d).

**Figure 7:**
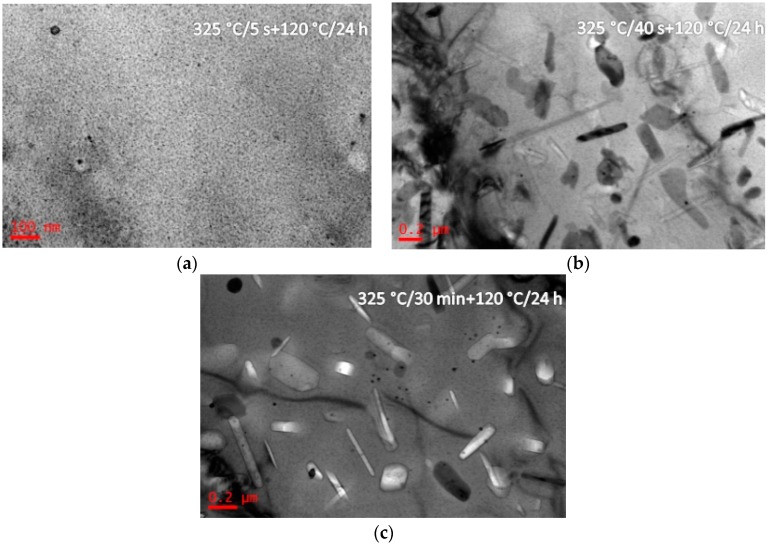
BF images of the studied alloy having been isothermal heat treated at 325 °C for different durations (5 s, 40 s, and 30 min), and then aged at 120 °C for 24 h. The isothermal heat treatment durations are: (**a**) 5 s; (**b**) 40 s; and, (**c**) 30 min.

**Figure 8:**
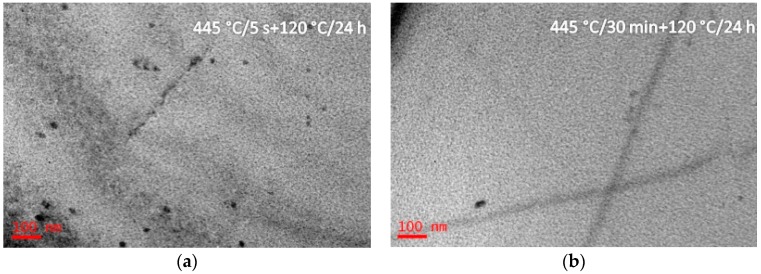
BF images of the studied alloy having been isothermal heat treated at 445 °C for different durations (5 s and 30 min), and then aged at 120 °C for 24 h. The isothermal heat treatment durations are: (**a**) 5 s; (**b**) 30 min.

Bright-field TEM images and SADPs of the solution treated alloy having been annealed at 325 °C for different durations (5 s, 40 s, and 30 min), and then aged at 120 °C for 24 h (Sample G) are shown in Figure 7. After isothermal heat treated at 325 °C for 5 s and aging at 120 °C for 24 h, η′ precipitates were formed with a size of 10–20 nm (Figure 7a). Increasing the IHT time to 40 s, coarse η′ precipitates were formed with a length of 300 nm and a diameter of 30 nm (Figure 7b). Increasing the IHT time to 30 min, η′ precipitates with a size of 300 nm in length and 100 nm in diameter (Figure 7c). Figure 8 shows BF images of the solution treated alloy having been isothermal heat treated at 445 °C for different durations (5 s and 30 min), and then aged at 120 °C for 24 h (sample H). After isothermal heat treated at 445 °C for 5 s and aged at 120 °C for 24 h, some η′ precipitates were detected with a size of 10–20 nm (Figure 8a), and they maintained same size even if isothermal heat treated for 30 min (Figure 8b). Al_3_Zr precipitates were also detected in these specimens (Figure 7 and Figure 8).

### 3.2. Hardness Variation

**Figure 9:**
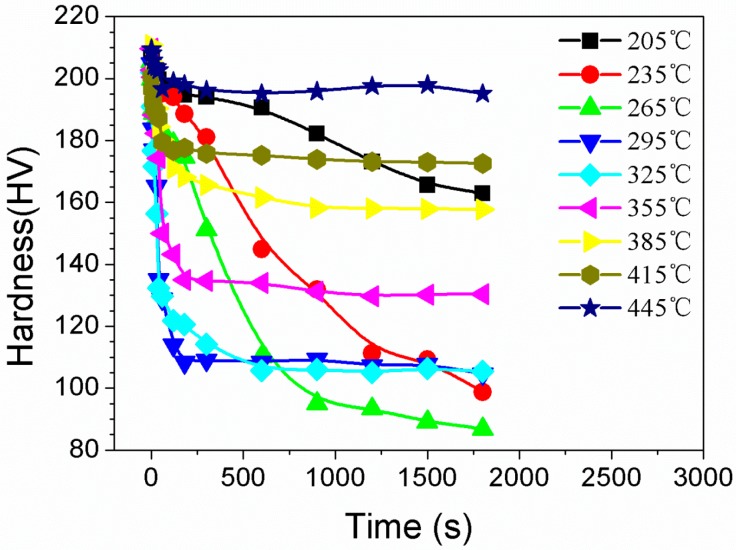
Hardness variation via the isothermal treated time on the studied samples having been solution treated at 470 °C for 1 h, isothermal heat treated for various durations, and then aged at 120 °C for 24 h.

The effect of IHT on the mechanical properties of the alloy was investigated through hardness measurements. Figure 9 shows the hardness variation versus the isothermally heat treated time on studied alloy samples having been solution treated at 470 °C for 1 h and quenched (cooling rate of 200 °C/s), isothermal heat treated at different temperatures for various durations, and then aged at 120 °C for 24 h. With increasing the IHT time, the hardness decreased successively in all IHT temperatures. Especially when the samples were isothermal heat treated at 295 °C (or 325 °C) for 3 min, the hardness values were rapidly decreased from 210 HV to 110 HV for 295 °C, and from 210 HV to 120 HV for 325 °C. However, when samples were isothermal heat treated at 205 °C (sample F), the hardness decreased slightly. The hardness decreased from 210 HV to 198 HV when the sample was isothermal heat treated at 205 °C for 3 min. When a specimen was solution treated at 470 °C for 1 h and then immediately aged at 120 °C for 24 h (sample A), the hardness was as high as 210 HV. The hardness of specimens decreased slightly from 210 HV to 202 HV when the SSS specimens were transferred into a furnace at the temperature of 445 °C and kept for 3 min, followed by aging at 120 °C for 24 h (sample H). As samples were transferred into the IHT furnace, kept warm in the furnace for 30 min, and then followed by aged at 120 °C for 24 h, the hardness of specimens was less than 160 HV. Further increasing the IHT time, hardness decreased successively.

### 3.3. Stress-Strain Curves

**Figure 10:**
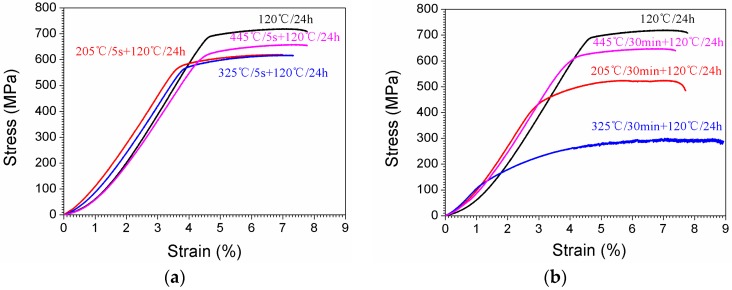
Stress-strain curves of the tensile samples having been isothermal heat treated at 205 °C, 325 °C, and 445 °C for different durations (5 s and 30 min), then aging at 120 °C for 24 h. (**a**) the isothermal heat treated time is 5 s; (**b**) the isothermal heat treated time is 30 min.

Figure 10 shows the tensile stress-strain curves of the tensile samples. For the solid solution treated sample directly aged at 120 °C for 24 h (sample A), the ultimate tensile strength was 719.8 MPa. While for samples having been isothermal heat treated at 205 °C, 325 °C, 445 °C, then aged at 120 °C for 24 h, the ultimate tensile strengths were 620.2 MPa (for 205 °C, sample F/5 s), 606.4 MPa (for 325 °C, sample G/5 s), and 647.5 MPa (for 445 °C, sample H/5 s), respectively. While for samples having been isothermal heat treated at 205 °C, 325 °C, 445 °C for 30 min, and then aged at 120 °C for 24 h, the ultimate tensile strength values were 524.5 MPa (for 205 °C, sample G/30 min), 300.1 MPa (for 325 °C, sample G/30 min), and 647.5 MPa (for 445 °C, sample G/30 min). Table 3 shows the detailed mechanical properties of the specimens with different heat treatments (see Table 2).

## 4. Discussion

### 4.1. The Quenching Sensitivity

**Figure 11:**
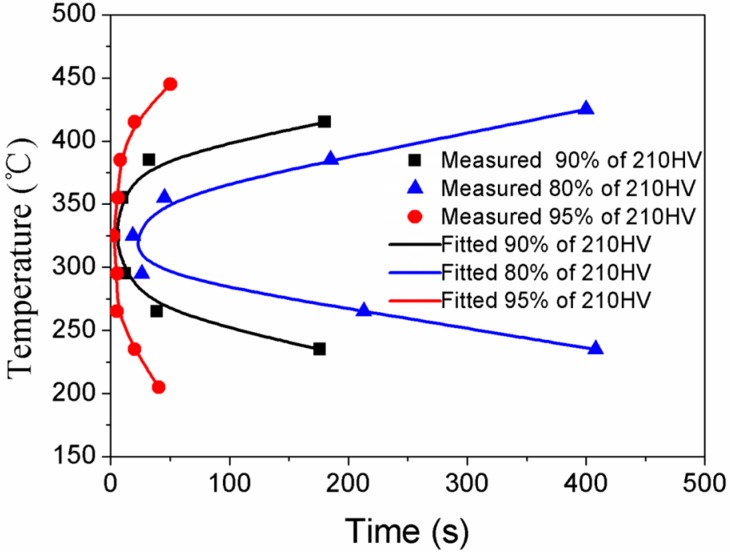
The time-temperature-property (TTP) curves of the studied alloy subjected to have been solid solution treated at 470 °C for 1 h, isothermal heat treatment for various durations, and then aged at 120 °C for 24 h.

Temperature-time-property (TTP) curves can be established by the hardness of the samples with isothermal heat treatment and aging treatment. The nucleation rate (*k*) can be expressed with the following equation [37,38]:

(1) $$k=\frac{t_{c}}{k_{1}}=k_{2}\exp[\frac{k_{3}k_{4}{}^{2}}{RT{\left(k_{4}-T\right)}^{2}}]\exp[\frac{k_{5}}{RT}]$$

where $t_{c}$ is the critical time required to precipitate a constant amount of solutes; *T* is the IHT temperature (degree K). *R* is the gas molar constant (8.31 J·K^−1^). *k*_1_, *k*_2_, *k*_3_, *k*_4_, and *k*_5_ are materials constants that are related to the natural logarithm of the volume fraction of untransformed precipitates (*k*_1_), the reciprocal of the number of nucleation sites (*k*_2_), the formation energy of nucleation sites (*k*_3_), solid solution temperature (*k*_4_), and the diffusion activation energy (*k*_5_). The fitted coefficients of TTP curves from the Figure 11 by the hardness data are 2.10 × 10^−11^ for *k*_2_, 1061 J/mol for *k*_3_, 802 K for *k*_4_, and 1.23 × 10^5^ J/mol for *k*_5_, respectively. TTP curves are “C” shape, whose nose temperatures are around 325 °C (Figure 11). 

For the studied alloy, we divided the temperature zone during the quenching process into low-temperature zone (<250 °C), medium-temperature zone (250–400 °C), and high-temperature zone (>400 °C). When the supersaturated solid solution were isothermal heat treated at the low-temperature zone (<250 °C) and high-temperature zone (>400 °C). The hardness decreased slightly. While, if SSS samples were isothermal heat treated at the medium-temperature zone (250–400 °C), the hardness decreased rapidly, indicating that the alloy presented a high quenching sensitivity and a short incubation period in the medium-temperature zone. Similar TTP curves of conventional casting 7XXX alloy have been reported by some researchers, and many methods were applied to study the quenching sensitivity. In-situ electrical resistivity measurement and differential calorimetry were employed to investigate the high temperature precipitation kinetics and TTT curves, which reveal that the nose temperature decreased from 375 °C to 350 °C when the precipitated fraction increase [37]. Liu et al. have investigated the TTP diagram for Al-Zn-Mg-Cu alloys including 7075, 7175, 7050, 7010, 7055, 7085, and 1933. Their result demonstrated that the nose temperature of TTP diagrams changed with the chemical composition of alloys, which was 355 °C for 7055 alloy while it was 295 °C for 7085 alloy [39]. Nie et al. reported that the aging hardness reductions for the 7085, 7050, 7150, and 7055 alloys by air quenching are up to 8.6%, 18.4%, 21%, and 21.7%, respectively, in comparison that that by water quenching [40]. Air quenching presents lower a quenching rate when compared with water quenching. The aging yield strength reductions for the studied alloy are 27.6%, 31.4%, and 11.2% for sample F/5 s, sample G/5 s, and sample H/30 min, respectively, in comparison that with water quenching, thus a higher furnace-to-furnace transfer temperature is preferred for the studied alloy. Due to recrystallization, Zr-containing dispersoids become incoherent with the matrix and act as effective nucleation sites for heterogeneous precipitation during slow quenching, thus Zr addition in the alloy inhibits recrystallization that is sensitive to the quench sensitivity [41]. The hardness of 7085 alloy increased with an increase of Mg, which was reported by Deng et al. who have studied the quench sensitivity of Al alloys via depth of age hardening layer according to hardness retention value [27]. The TTP curve of 7010 alloy indicated that transformation occurred most rapidly between 250 and 400 °C [42]. For the above 7XXX alloy, the nose temperature of TTP curves were between 250–400 °C, which are consistent with the result in this work (325 °C).

As the SSS was isothermal heat treated, some precipitates have been detected in above images and previous investigations [37,43,44]. High quenching sensitivity is the result of fast precipitation from the supersaturated solid solution [24]:

(2) $$\frac{dVt}{dt}=\frac{4}{3}\pi \cdot V_{0}\cdot \overset{•}{N}\cdot \overset{•}{G^{3}}\cdot t^{3}\cdot \exp(-\frac{\pi }{3}\overset{•}{G^{3}}\overset{•}{N}t^{4})$$

where *V*_t_ is the precipitates volume, *V*_0_ is the sample volume, $\overset{•}{N}$ is the nucleating rate of precipitates, $\overset{•}{G^{3}}$ is the precipitate growing rate, and *t* is IHT time. When the SSS samples were isothermal heat treated in the medium temperature zone (250–400 °C), $\overset{•}{N}\cdot \overset{•}{G^{3}}$ reached the maximum value. The incubation period of the studied alloy is short in medium temperature zone but long in the low-temperature zone and high-temperature zone. Thus, for the studied alloy, after solid solution treatment, the samples should be rapidly transferred into the aging treatment furnace, and the temperature of the sample during transfer process should be higher than 400 °C. Meanwhile, if the sample could not be moved to the aging furnace very quickly, a water quenching should be performed for cooling down the sample temperature to lower than 250 °C. Otherwise, the SSS sample would be decomposed and coarse precipitates would form on grain boundary and grain inside, resulting in undesired mechanical properties.

### 4.2. Precipitation Behavior during Isothermal Heat Treatment

The studied alloy was a precipitation hardening aluminum alloy. After the appropriate solution annealing and quenching, a supersaturated solid solution would be formed; then decomposition behavior would start from quenching, as well as aging at a low temperature. Previous investigations proved that the SSS would begin to decompose at 120 °C [45]. Generally, for the Al-Zn-Mg-Cu-Zr alloy, it will take 12 h to finish the peak aging process [43]. The precipitates size was only 5–8 nm in length and 1.5–3 nm in width. With increasing the aging temperature, the decomposition behavior accelerated [46]. During the isothermal heat treatment processing, few η′ precipitates were detected when the IHT time is only 5 s (Figure 3b). Increasing the time to IHT 30 min, some precipitates with a size of 80 nm in length and 10 nm in diameter were detected (Figure 3d). However, when the SSS was quenched to isothermal temperature of 325 °C (Figure 4), even the IHT time is 5 s, some η′ precipitates were detected with a size of 150 nm in length and 20 nm in diameter (Figure 4a). Similarly, the SSS sample was quenched to an isothermal temperature of 445 °C, precipitation happened (Figure 5). These isothermal heat treated specimens were aging treated at 120 °C for 24 h, many η′-MgZn_2_ were precipitated from the matrix. As a result of this work, an appropriate isothermal temperature required to be tailored, low-temperature zone (<250 °C) and high-temperature zone (>400 °C) were worked out for the studied alloy to maintain small precipitates and high strength.

### 4.3. Influence of Precipitates Size on the Strengthening Response

Low-temperature aging treatment at 120 °C contributed to the precipitation hardening in the studied alloy. However, the precipitation occurred in samples during transferring from the solution treated furnace to the aging furnace, and the precipitates may coarsen. The Orowan mechanism for dislocation and particles can evaluate the significant increase of yield strength by the precipitates. The increment of Δ*σ*_Orowan_ can be expressed as follows [36].

(3) $$\Delta \sigma \;=\;\frac{0.81MGb}{2\pi {\left(1-\upsilon \right)}^{1/2}}\frac{\ln\left(d_{p}/b\right)}{\left(\lambda -d_{p}\right)}$$

(4) $$\lambda \;=\frac{1}{2}d_{p}\sqrt{\frac{3\pi }{2f_{v}}}$$

where, *M* is the Taylor-factor (*M* for Al is 3.06); *G* is the shear modulus (shear modulus for Al is 26.9 GPa); *d*_p_ is the average diameter of precipitates particle; *b* is the Burgers vector (*b* for Al is 0.286 nm); *ν* is the Poisson's ratio (*ν* for Al is 0.33) [47]; *λ* is the average particle plane square lattice spacing; *f*_v_ is the volume fraction of particles. To simplify the calculation, we assumed that all of the Mg and Zn atoms precipitated in the form of η′-MgZn_2_ precipitates [48]. The volume fraction of MgZn_2_ precipitates can be calculated and *f*_v_ is 5.9%. Therefore, the increment of Δ*σ*_Orowan_ was a function of *d*_p_ and can be calculated as follows:

(5) $$\Delta \sigma_{\mathrm{Orowan}}=\frac{1286+1029\ln dp}{dp}$$

When the sample was solution-treated and immediately aged at 120 °C for 24 h, *d*_p_ was about 10 nm (Figure 2, sample B), the Δ*σ*_Orowan_ was 365.5 MPa. However, when the *d*_p_ was about 300 nm in Figure 7c (the alloy was isothermal treated at 325 °C for 30 min then aged at 120 °C for 24 h, sample G), the Δ*σ*_Orowan_ is only 23.9 MPa. The precipitate size is a significant factor on the Δ*σ*_Orowan_ strengthening effect.

## 5. Conclusions

In summary, a high strength Al-Zn-Mg-Cu-Zr alloy was synthesized by using a spray deposition technology followed by hot extrusion, solution treatment, isothermal heat treatment, and aging treatment.

(1) The hardness and the ultimate tensile strength decreased with the isothermal heat treatment time. TTP curves of the studied alloy have been established, and the mechanism for high quenching sensitivity at the nose temperature zone has been discussed. TTP curves of the studied alloy are “C”-like ones, and the nose-tip temperatures of these curves are around 325 °C.
(2) The studied alloy presented a high quenching sensitivity at the medium-temperature zone (250–400 °C) because in this medium temperature zone the nucleation rate of the precipitates was high and a significant number of precipitates formed. After solid solution treatment, precipitation appeared scarcely as specimens had been isothermal heat treated at the low-temperature zone (<250 °C), and the high-temperature zone (>400 °C), both of above two temperature zones were available for the industry production.
(3) The strengthening phase would precipitate as the sample aged at 120 °C, resulting in high mechanical properties. Precipitates with size of 10 nm would contribute a significant increase in yield strength, while ones larger than 300 nm contribute only little.

## Figures and Tables

**Table 1:** Chemical composition of studied Al-Zn-Mg-Cu-Zr alloy samples.

Composition	Al	Zn	Mg	Cu	Zr	Fe	Si	Ti	Mn	Cr
wt %	Bal.	8.31	2.07	2.46	0.12	0.078	0.056	0.005	0.005	0.003
at %	Bal.	3.68	2.45	1.12	0.038	0.04	0.057	0.003	0.003	0.002

**Table 2:** Heat treatment parameters of the samples with different methods.

Sample No.	Solution Treatment	Isothermal Heat Treatment	Aging Treatment
A	470 °C/1 h	/	/
B	470 °C/1 h	/	120 °C/24 h
C	470 °C/1 h	205 °C/5 s (30 min)	/
D	470 °C/1 h	325 °C/5 s (40 s), (30 min)	/
E	470 °C/1 h	445 °C/5 s, (30 min)	/
F	470 °C/1 h	205 °C/5 s (30 min)	120 °C/24 h
G	470 °C/1 h	325 °C/5 s (40 s), (30 min)	120 °C/24 h
H	470 °C/1 h	445 °C/5 s (30 min)	120 °C/24 h

**Table 3:** Mechanical properties of the studied specimens subjected to isothermal heat treatments for different treatment parameters.

Sample No.	Elongation (%)	Ultimate Tensile Strength (MPa)	Yield Strength (MPa)
B	7.7	719.8	699.8
C/5 s	6.9	620.2	598.2
D/5 s	7.3	604.4	578.3
E/5 s	7.7	647.5	621.9
F/30 min	7.7	524.5	506.4
G/30 min	8.9	300.1	267.6
H/30 min	7.3	647.5	621.2
